# Whole-genome long-read TAPS deciphers DNA methylation patterns at base resolution using PacBio SMRT sequencing technology

**DOI:** 10.1093/nar/gkac612

**Published:** 2022-07-18

**Authors:** Jinfeng Chen, Jingfei Cheng, Xiufei Chen, Masato Inoue, Yibin Liu, Chun-Xiao Song

**Affiliations:** Ludwig Institute for Cancer Research, Nuffield Department of Medicine, University of Oxford, Oxford OX3 7FZ, UK; Target Discovery Institute, Nuffield Department of Medicine, University of Oxford, Oxford OX3 7FZ, UK; Ludwig Institute for Cancer Research, Nuffield Department of Medicine, University of Oxford, Oxford OX3 7FZ, UK; Target Discovery Institute, Nuffield Department of Medicine, University of Oxford, Oxford OX3 7FZ, UK; Ludwig Institute for Cancer Research, Nuffield Department of Medicine, University of Oxford, Oxford OX3 7FZ, UK; Target Discovery Institute, Nuffield Department of Medicine, University of Oxford, Oxford OX3 7FZ, UK; Ludwig Institute for Cancer Research, Nuffield Department of Medicine, University of Oxford, Oxford OX3 7FZ, UK; Target Discovery Institute, Nuffield Department of Medicine, University of Oxford, Oxford OX3 7FZ, UK; Ludwig Institute for Cancer Research, Nuffield Department of Medicine, University of Oxford, Oxford OX3 7FZ, UK; Target Discovery Institute, Nuffield Department of Medicine, University of Oxford, Oxford OX3 7FZ, UK; Ludwig Institute for Cancer Research, Nuffield Department of Medicine, University of Oxford, Oxford OX3 7FZ, UK; Target Discovery Institute, Nuffield Department of Medicine, University of Oxford, Oxford OX3 7FZ, UK

## Abstract

Long-read sequencing provides valuable information on difficult-to-map genomic regions, which can complement short-read sequencing to improve genome assembly, yet limited methods are available to accurately detect DNA methylation over long distances at a whole-genome scale. By combining our recently developed TET-assisted pyridine borane sequencing (TAPS) method, which enables direct detection of 5-methylcytosine and 5-hydroxymethylcytosine, with PacBio single-molecule real-time sequencing, we present here whole-genome long-read TAPS (wglrTAPS). To evaluate the performance of wglrTAPS, we applied it to mouse embryonic stem cells as a proof of concept, and an N50 read length of 3.5 kb is achieved. By sequencing wglrTAPS to 8.2× depth, we discovered a significant proportion of CpG sites that were not covered in previous 27.5× short-read TAPS. Our results demonstrate that wglrTAPS facilitates methylation profiling on problematic genomic regions with repetitive elements or structural variations, and also in an allelic manner, all of which are extremely difficult for short-read sequencing methods to resolve. This method therefore enhances applications of third-generation sequencing technologies for DNA epigenetics.

## INTRODUCTION

In mammalian genomes, DNA cytosine modifications including canonical 5-methylcytosine (5mC), 5-hydroxymethylcytosine (5hmC), 5-formylcytosine and 5-carboxylcytosine (5caC) are the key epigenetic mechanisms for regulating spatiotemporal gene expression (1). As the predominant epigenetic marks, the functional roles of 5mC and 5hmC in normal mammalian development and human diseases have been thoroughly interrogated (2–5). Over recent decades, short-read next-generation sequencing has been the predominant method for genomic research (6). Conventional short-read bisulfite sequencing (BS-seq) is still regarded as the gold standard for methylome analysis (7). This method is based on the treatment of DNA with sodium bisulfite. Unmodified cytosines are deaminated to uracils, while 5mC and 5hmC remain intact. Uracil residues are then read as thymine (T) during PCR amplification resulting in C-to-T conversion of unmodified cytosines (8). Despite its widespread adoption, BS-seq suffers from two main drawbacks. First, it employs a harsh chemical reaction, which causes severe DNA degradation due to depyrimidination of DNA (9). This issue makes it difficult to obtain sufficiently long DNA fragments for long-read sequencing. Indeed, the longest PCR amplicons obtained and sequenced from bisulfite-treated DNA do not exceed 1.5 kb in length (10). Second, bisulfite libraries have reduced sequence complexity caused by C-to-T conversion of unmodified cytosines, which accounts for ∼95% of all cytosines in the human genome. Reduced complexity results in poor sequencing quality, low mapping rate and uneven genome coverage (11).

To circumvent these limitations, two bisulfite-free approaches have recently been developed. The first one, from our laboratory, is TET-assisted pyridine borane sequencing (TAPS) (12). In the TAPS method, 5mC and 5hmC are oxidized by ten-eleven translocation (TET) proteins to 5caC and then reduced to dihydrouracil (DHU) by pyridine borane. DHU is subsequently amplified and sequenced as T during sequencing. TAPS is non-destructive and shows better sequencing quality, mapping rate and coverage compared to BS-seq (12). The second method, enzymatic methyl sequencing (EM-seq), also employs TET to convert 5mC into 5caC. Simultaneously, 5hmC is converted to 5-(β-glucosyloxymethyl)cytosine using T4 phage β-glucosyltransferase. Next, apolipoprotein B mRNA editing enzyme catalytic polypeptide-like 3A deaminates cytosines into uracils, but not the protected forms of 5mC or 5hmC (13). Compared to TAPS, although EM-seq overcomes the issue of DNA damage from BS-seq, it still suffers from the indirect detection through converting unmodified cytosine to thymine, which leads to the same low-complexity problem in the resulting sequencing library (14).

In recent years, the two dominant long-read sequencing techniques have been single-molecule real-time (SMRT) sequencing by Pacific Biosciences (PacBio) and Nanopore sequencing by Oxford Nanopore Technologies (15,16). Long-read sequencing techniques hold the promise to access complex regions of the genome, offering unprecedented opportunities for accurately assembling genomes (17), phasing haplotypes (18), sequencing of tandem repeats (19), resolving structural variants over large tracts (20) and studying of genomic imprinting (21). DNA base modifications can affect the kinetics of the polymerase during SMRT sequencing (22) or alter the electric current patterns in Nanopore sequencing, respectively (23). Therefore, in principle, SMRT and Nanopore platforms can directly sequence DNA samples without PCR amplification or additional base conversion. However, in practice, SMRT sequencing requires ultra-high sequence coverage (up to 250×) for the detection of 5mC (24), and Nanopore sequencing requires complicated training data set from control samples with known methylation state as well as complex computational analysis (24). Furthermore, both SMRT and Nanopore DNA methylation sequencing require microgram levels of native nonamplified DNA as input. These barriers significantly hinder applications of long-read sequencing for DNA epigenetics. Given that both TAPS and EM-seq methods better preserve longer DNA fragments under much milder enzymatic and chemical conditions, combining long-read sequencing and bisulfite-free approaches would overcome the above-mentioned limitations and further provide highly accurate long-read epigenetic sequencing. Indeed, targeted long-read TAPS (lrTAPS) and long-read EM-seq were capable of precisely profiling long-range methylation over multikilobase lengths of genomic DNA (gDNA) (24,25). Furthermore, the efficacy of whole-genome long-read methylome analysis using Oxford Nanopore sequencing and EM-seq (nanoEM) was recently studied (26). However, nanoEM has a relatively low mapping rate, and still involves indirect methylation detection.

To further expand the application of long-read epigenetic sequencing, we here present another novel approach for whole-genome long-read methylome detection at base-level resolution through the combination of the TAPS method and PacBio SMRT sequencing [herein termed whole-genome long-read TAPS (wglrTAPS)]. The latest PacBio SMRT sequencing enables read sequencing accuracies >99.9% (27). In contrast, Nanopore sequencing usually achieves <90% accuracy (15). In this work, we applied wglrTAPS to gDNA from mouse embryonic stem cells (mESCs) and compared it with our short-read TAPS. We showed that wglrTAPS can cover challenging regions and simultaneously detect the methylation status on those regions.

## MATERIALS AND METHODS

### Preparation of spike-in control

A 4-kb DNA spike-in control was prepared by PCR amplification of the pNIC28-Bsa4 plasmid (Addgene, cat. no. 26103) in a reaction containing 1 ng DNA template, 0.5 μM primers and 1× Phusion High-Fidelity PCR Master Mix with HF Buffer (Thermo Scientific). Primer sequences are listed in Supplementary Table S1. Thermal cycling consisted of 1 cycle of 98°C for 30 s and 25 cycles of 98°C for 10 s, 62°C for 15 s and 72°C for 63 s, followed by final extension at 72°C for 10 min and then hold at 4°C. The PCR product was purified by Zymo-IC column (Zymo Research) with Buffer PB (Qiagen), and the concentration was measured using Qubit dsDNA HS Assay Kit (Thermo Fisher) followed by 1% agarose gel electrophoresis to check purity. The purified PCR product up to 1 μg was then methylated at 37°C for 2 h in a 50 μl reaction with 32 nmol of SAM and 10 U of HpaII methyltransferase (New England Biolabs) in 1× CutSmart buffer. Then, 1.25 μl of HpaII methyltransferase (4 U/μl) and 1 μl of SAM (32 mM) were added to the reaction and incubated at 37°C for another 2 h after which the C^m^CGG methylated product was purified using 1× AMPure XP beads (Beckman Coulter) according to the manufacturer’s protocol. The efficiency of DNA methylation was verified by HpaII digestion assay. Fifty nanograms of unmethylated and methylated DNA was digested in a 10 μl reaction with 2 U of HpaII restriction endonuclease (New England Biolabs) in 1× CutSmart buffer at 37°C for 1 h.

### Expression and purification of mTet1CD

The production of recombinant mTet1CD was prepared as described previously (12) with a few modifications. The mTet1CD catalytic domain (NM_001253857.2, 4371–6392) with N-terminal Flag-tag was cloned into pcDNA3-Flag between the KpnI and BamH1 restriction sites. For protein expression, 1 mg plasmid was transfected into 1 l of Expi293F cell culture at a density of (1.5–2) × 10^6^ cells/ml and cells were grown for 48 h at 37°C, 170 rpm and 6% CO_2_. Subsequently, cells were harvested by centrifugation at 1500 × *g* and 4°C for 20 min, resuspended in the lysis buffer containing 50 mM Tris–Cl (pH 7.5), 500 mM NaCl, 1× cOmplete Protease Inhibitor Cocktail and 1% Triton X-100 on a magnetic stirrer for 20 min at 4°C. Cell lysate was then clarified by centrifugation for 1 h at 35 000 × *g* and 4°C. Collected supernatant was purified on ANTI-FLAG M2 Affinity Gel and pure protein was eluted with buffer containing 20 mM HEPES (pH 8.0), 150 mM NaCl, 0.1 mg/ml 3× Flag peptide and 1× cOmplete Protease Inhibitor Cocktail. Collected fractions were concentrated and buffer exchanged to the final buffer containing 20 mM HEPES (pH 8.0), 150 mM NaCl and 1 mM dithiothreitol. Concentrated protein was mixed with glycerol (30%, v/v), frozen in liquid nitrogen and aliquots were stored at −80°C.

### mESC culture and isolation of gDNA

E14 mESCs were cultured on gelatin-coated plates in DMEM (Invitrogen) supplemented with 15% FBS (Gibco), 2 mM l-glutamine (Gibco), 1% non-essential amino acids (Gibco), 1% penicillin/streptavidin (Gibco), 0.1 mM β-mercaptoethanol (Sigma), 1000 units/ml leukaemia inhibitory factor (Millipore), 1 μM PD0325901 (Stemgent) and 3 μM CHIR99021 (Stemgent). Cultures were maintained at 37°C and 5% CO_2_. For isolation of gDNA, cells were harvested by centrifugation for 5 min at 1000 × *g* and room temperature. DNA was extracted with Quick-DNA Plus Kit (Zymo Research) according to the manufacturer’s protocol.

### mTet1CD oxidation

Genomic DNA (up to 100 ng) was incubated in a 25 μl reaction containing 50 mM HEPES buffer (pH 8.0), 100 μM ammonium iron(II) sulfate, 1 mM α-ketoglutarate, 2 mM l-ascorbic acid, 2.5 mM dithiothreitol, 1.2 mM ATP, 100 mM NaCl and 4 μM mTet1CD at 37°C for 80 min. After that, 0.8 U of Proteinase K (New England Biolabs) was added to the reaction mixture and incubated at 50°C for 1 h. The oxidation reaction was purified by 1× AMPure XP beads following the manufacturer’s instructions. To achieve complete oxidation, second round of oxidation was performed following the steps as described earlier. The double-oxidized gDNA was then eluted into nuclease-free water.

### wglrTAPS

mESC gDNA was fragmented to 5 kb using miniTUBE Red (Covaris) according to the manufacturer’s protocol. Size selection of the sheared gDNA was then performed to remove the short fragments (<3 kb) using a 3.7× ratio of diluted AMPure XP beads:sample. For preparation of the diluted beads, AMPure XP beads were diluted with elution buffer to a final 35% (v/v) and stored at 4°C before use. Next, fragmented and size-selected gDNA spiked-in with 0.5% of C^m^CGG methylated 4-kb control DNA was end-repaired and dA-tailed, after which ligation of Y-shaped adapter was prepared using KAPA Hyper Kit according to the manufacturer’s protocol. The sequences of Y-shaped adapter are listed in Supplementary Table S1, and pre-annealed before use. Briefly, 15 μl of 100 μM oligonucleotides (IDT, HPLC purified) were annealed in the annealing buffer containing 10 mM Tris–Cl (pH 7.9), 0.1 mM EDTA (pH 8.0) and 50 mM NaCl. The adapter-ligated sample was then double oxidized by mTet1CD as described earlier. Oxidized DNA sample in 42.5 μl of water was reduced in a 50 μl reaction using optimized borane reduction conditions at 37°C and 850 rpm for 4 h in an Eppendorf ThermoMixer. Subsequently, 15 ng of TAPS-converted DNA was amplified using barcoded primers (sequences are listed in Supplementary Table S1) and LongAmp Hot Start Taq 2× Master Mix (New England Biolabs). Thermal cycling consisted of 1 cycle of 94°C for 30 s and 16 cycles of 94°C for 15 s, 60°C for 30 s and 65°C for 5 min 20 s, followed by final extension at 65°C for 10 min and then hold at 4°C. The amplified DNA was purified using 0.4× AMPure XP beads. Seven hundred fifty nanograms of amplified DNA was then used to construct HiFi SMRTbell library with a SMRTbell Express Template Prep Kit 2.0 (Pacific Biosciences) following the manufacturer’s instructions. For sequencing, the SMRTbell library was bound with Sequel II Binding Kit 2.0, sequenced with Sequel II Sequencing Plate 2.0 using a 30-h movie with 1 h pre-extension time. ‘Application’ was set to ‘≥3 kb amplicons’. Sequencing data were demultiplexed and CCS reads computed using the SMRT Analysis package (Pacific Biosciences) with minimum three passes and minimum predicted accuracy = Q20.

### Data analysis for wglrTAPS and short-read TAPS

For wglrTAPS, CCS reads were classified into two groups according to their ligated adapter using cutadapt 1.18 (28) with the following parameters: reads trimmed with -g CCGAGATCTACACTCTTTCCCTACACGACGCTCTTCCGATCT -a AGATCGGAAGAGCACACGTCTGAACTCCAGTCACCGATGTATCTCGTA were classified as forward strand, and the remaining reads trimmed with -g TACGAGATACATCGGTGACTGGAGTTCAGACGTGTGCTCTTCCGATCT -a AGATCGGAAGAGCGTCGTGTAGGGAAAGAGTGTAGATCTCGG were classified as reverse strand. Trimmed reads were then aligned to the reference sequence using minimap2 2.16-r922 (29) with -a -x map-pb option. mm9 genome was used as reference sequence, which was downloaded from http://hgdownload.cse.ucsc.edu/goldenpath/mm9/bigZips/. Alignment files were then split into four groups according to their flag and read group with samtools 1.11 (30). Forward strand reads with flag = 16 and reverse strand reads with flag = 0 come from the OT strand, so their flag was assigned as 0. Reverse strand reads with flag = 16 and forward strand reads with flag = 0 come from the OB strand, so their flag was assigned as 16. Processed alignment files were then merged, and PCR duplicates were called using Picard (2.23.0-Java-11) MarkDuplicates (https://broadinstitute.github.io/picard/). Reads with mapping quality <10 were excluded for methylation calling. Modified bases were then called using MethylDackel 0.5.1 (https://github.com/dpryan79/MethylDackel). MethylDackel was designed for BS-seq, which converts unmodified cytosine to thymine. Therefore, we took the output of MethylDackel and used the ratio between the number of unmethylated bases and (number of unmethylated bases + number of methylated bases) to get the modification level for each cytosine. The repeat regions were downloaded from http://www.repeatmasker.org/genomes/mm9/RepeatMasker-rm328-db20090604/mm9.fa.out.gz. The CpG island track was downloaded from http://hgdownload.soe.ucsc.edu/goldenPath/mm9/database/cpgIslandExt.txt.gz. The gene annotation file was downloaded from http://hgdownload.soe.ucsc.edu/goldenPath/mm9/database/refGene.txt.gz. CpG sites (TCGG/CCGT/ACGG) that are more likely to be off-targets of HpaII methyltransferase were excluded when calculating the false-positive rate on 4-kb spike-in (24).

For short-read TAPS and BS-seq for mESCs, published data (GSE112520) were processed as described earlier (12). Samtools 1.11 were used to downsample short-read TAPS to the same depth as wglrTAPS.

### SV detection

For structural variation (SV) analysis in long reads, we ran cuteSV 1.0.11 (31) with the following parameters: --max_cluster_bias_INS 1000 --diff_ratio_merging_INS 0.9 --max_cluster_bias_DEL 1000 --diff_ratio_merging_DEL 0.9 --min_support 3. For SV analysis in short-read TAPS, we used Manta 1.6.0 (32) with minScoredVariantSize = 50. To find the SV identified in both wglrTAPS and short-read TAPS, we first extend the SV to its flanking 5 bp and then identify the overlap between the two SV sets using bedtools (33) intersect. SV regions were then visualized using Integrative Genomics Viewer (IGV) (34) in bisulfite mode.

## RESULTS

### Construction of wglrTAPS library and PacBio SMRT sequencing

**Figure 1.**
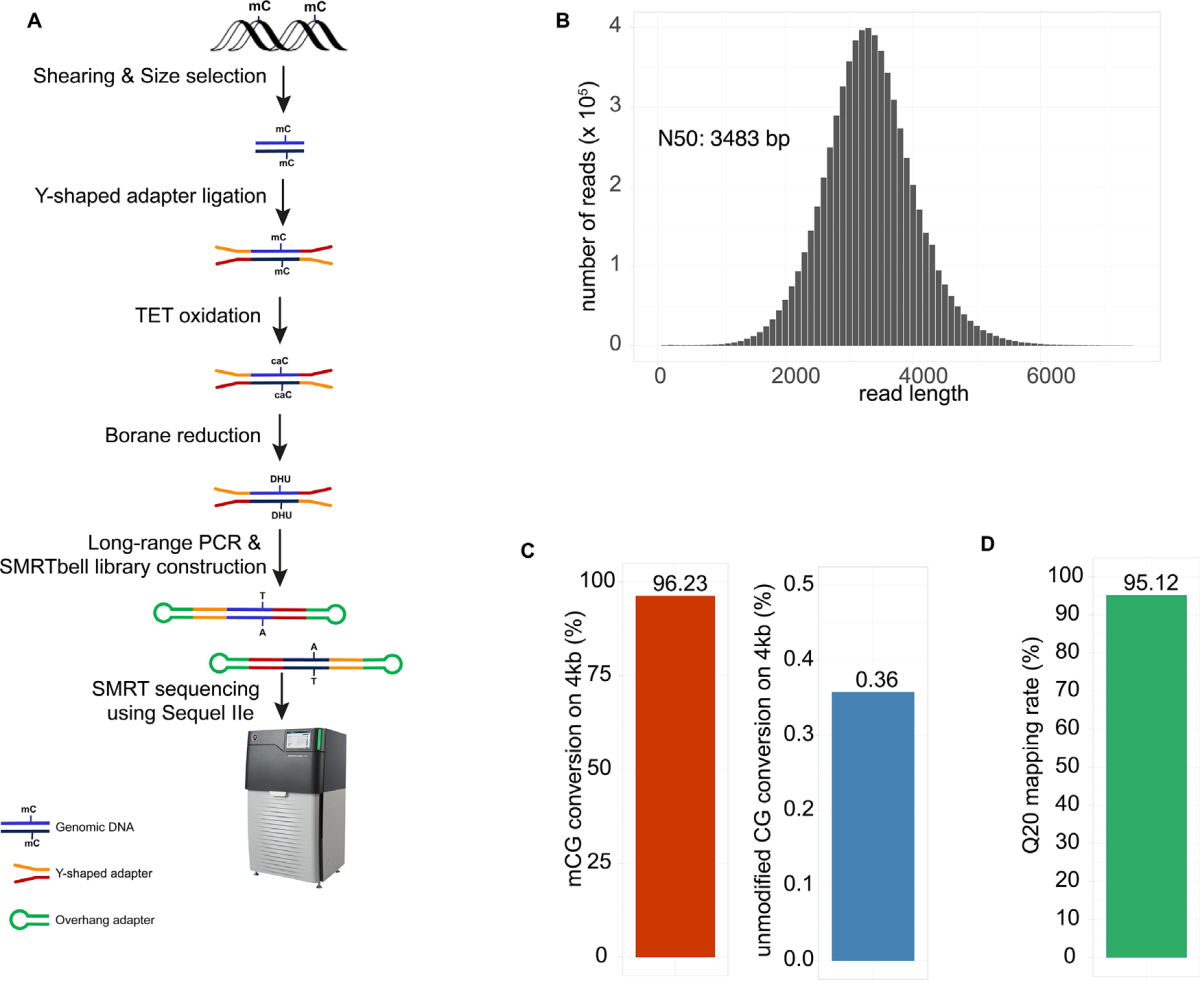
Development of wglrTAPS. (**A**) Schematic representation of the wglrTAPS. (**B**) Sequence length distribution of wglrTAPS HiFi read. (**C**) Conversion rate of wglrTAPS at methylated CpG sites and false-positive rate of wglrTAPS at non-methylated CpG sites from C^m^CGG-methylated 4-kb spike-in. (**D**) Fraction of mapped reads with ≥Q20 in wglrTAPS.

To implement wglrTAPS, we devised a novel procedure for library preparation depicted in Figure 1A. In this strategy, intact mESC gDNA was first fragmented to 5 kb in length using miniTUBE. In order to effectively eliminate short DNA fragments (<3 kb), size selection was conducted using diluted AMPure XP beads (see the ‘Materials and Methods’ section). Next, end repair and Y-shaped adapter ligation were performed, which allows strand-specific methylation detection. Following mTet1CD oxidation, the DNA was subjected to an optimized borane reduction, which improved long-range amplification (data not shown). TAPS-converted gDNA was then PCR amplified and subjected to preparation of final SMRTbell library, which was then sequenced using PacBio Sequel IIe.

To evaluate the reproducibility of this method, two technical library replicates were prepared and sequenced at different depths (replicate 1: 1.0×; replicate 2: 7.2×). As shown in Supplementary Figure S1A, a steady conversion rate (96.21% versus 96.31%) and false-positive rate (0.36% versus 0.37%) were achieved, and a consistent methylation profile (Pearson’s *r* for 500-kb bins: 0.84; Supplementary Figure S1B) was observed, which demonstrated a good reproducibility of this method. For the following analysis, we combined these two technical replicates to obtain 6.5 million reads or 21.8 Gb in total read bases (equal to 8.2× of mouse genome) with the size of the DNA fragments ranging between 51 and 14 115 bp. The N50 read length was 3483 bp (Figure 1B). To evaluate the performance of wglrTAPS, we used a 4-kb spike-in control DNA with all CpGs in CCGG sites methylated by HpaII methyltransferase as C^m^CGG. High 5mC conversion rate (96.23% in modified CpG) and low false-positive rate (0.36% conversion rate on unmodified CpG and 0.12% on unmodified CpH) were achieved in wglrTAPS (Figure 1C), which are comparable to standard short-read TAPS (12). Compared with nanoEM, which requires a specially designed alignment pipeline (26), wglrTAPS can be processed by the use of the standard long-read aligner—minimap2 (29), followed by a customized modification caller based on MethylDackel (see the ‘Materials and Methods’ section). This dramatically simplifies the downstream data analysis. As the TAPS method detects modified cytosine directly, wglrTAPS can indeed achieve higher mapping rate (95.12% Q20 mapping rate) (Figure 1D). In comparison, optimized nanoEM protocol has a mapping rate of 89–90% (26), and short-read TAPS achieves a unique mapping rate of 88.08% (12).

### Evaluation of wglrTAPS on mESCs

**Figure 2.**
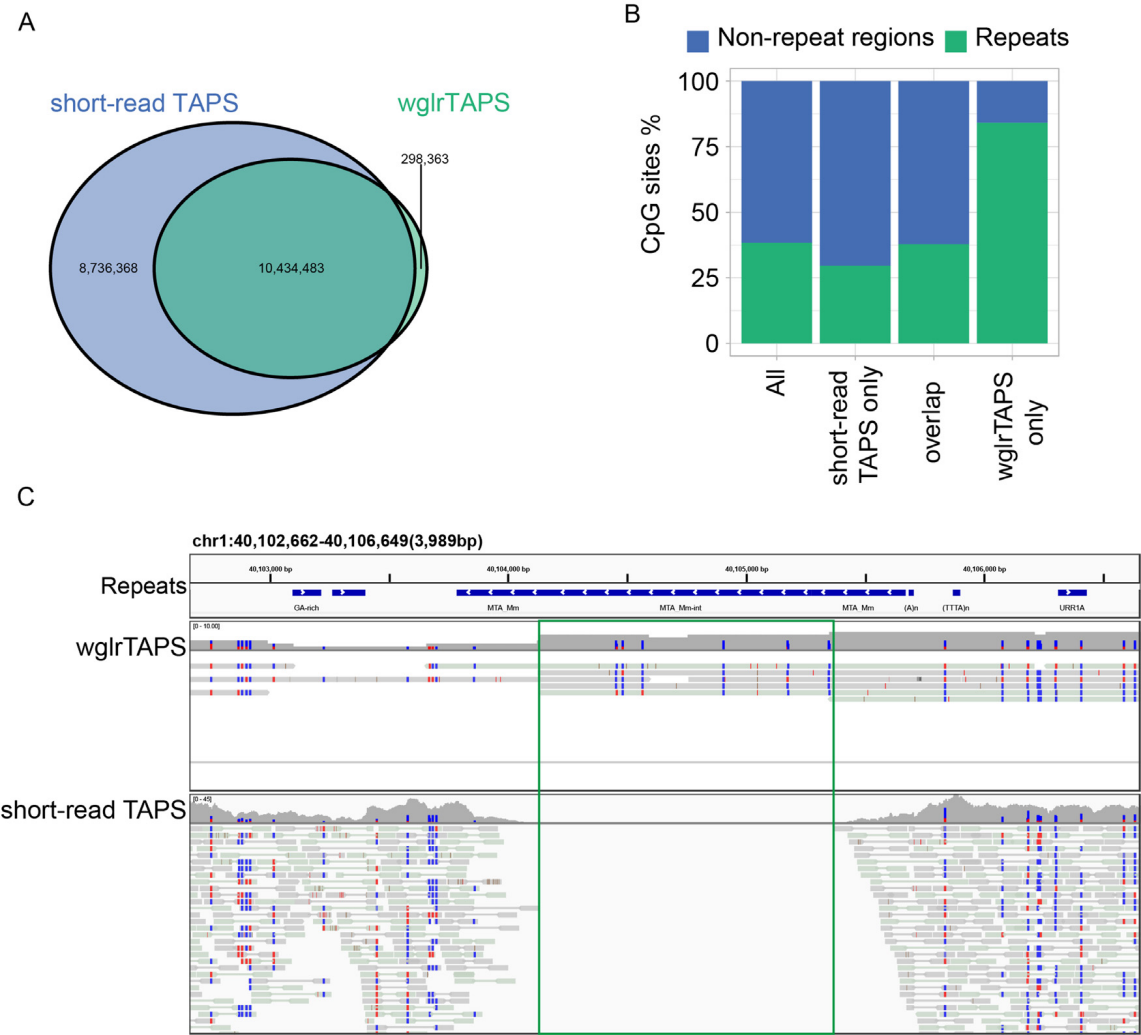
Comparison of covered CpG between short-read TAPS and wglrTAPS. (**A**) Venn diagram showing the number of CpG sites that were covered in short-read TAPS and wglrTAPS; CpG sites with at least five reads covered were used for calculation. (**B**) Bar plot showing the fraction of CpG sites overlapping with repeat regions in mouse genome. (**C**) An example of a repeat region that is only covered in wglrTAPS visualized using IGV in bisulfite mode with CG option. The blue colour denotes converted cytosine, and the red colour denotes unconverted cytosine. The top panel shows alignments from wglrTAPS; the bottom panel shows alignments from short-read TAPS. The repeat region that is only covered in wglrTAPS is highlighted in green box.

To further assess the performance of wglrTAPS, we compared the sequencing results with those obtained from short-read TAPS (12). Of the 20 996 248 CpG sites of the mouse genome, wglrTAPS and short-read TAPS (depth ≥ 5) covered 10 732 846 and 19 170 851 CpG sites, respectively (Figure 2A). The number of overlapping CpG sites was 10 434 483. We further examined the overlap between repeat regions and CpG sites. As shown in Figure 2B and C, a large proportion of CpG sites that were solely covered by wglrTAPS tended to locate in repeat regions of the genome. This result is consistent with previous findings that long-read sequencing can cover repetitive elements more efficiently than short-read sequencing, which suffers from ambiguous mapping at low-complexity regions (15). In addition, a good correlation was observed between wglrTAPS and short-read TAPS (Pearson’s *r* = 0.68; Supplementary Figure S2A). The discrepancy between them is likely caused by relatively low sequencing depth of wglrTAPS (8.2× sequencing depth). As we expected, the correlation increased to 0.76 when only CpGs sites covered by at least 20 reads were taken into consideration (Supplementary Figure S2B). Upon further interrogation on methylation patterns around gene body, a similar pattern was observed between wglrTAPS and short-read TAPS (Supplementary Figure S2C). CpG islands are known to lack DNA methylation, and this is reflected in both scenarios (Supplementary Figure S2D). In terms of methylation level, wglrTAPS showed slightly lower methylation compared to short-read TAPS, which could suggest that wglrTAPS generally covers lowly methylated regions with higher efficiency. The same phenomenon was also observed for nanoEM (26). To rule out the effect of different DNA treatments, we conducted further comparison between wglrTAPS and BS-seq. As shown in Supplementary Figure S2E and F, a good correlation was also observed between wglrTAPS and BS-seq (Pearson’s *r* for CpGs with depth ≥ 5: 0.63; Pearson’s *r* for CpGs with depth ≥ 20: 0.69).

### Detecting SV using wglrTAPS

**Figure 3.**
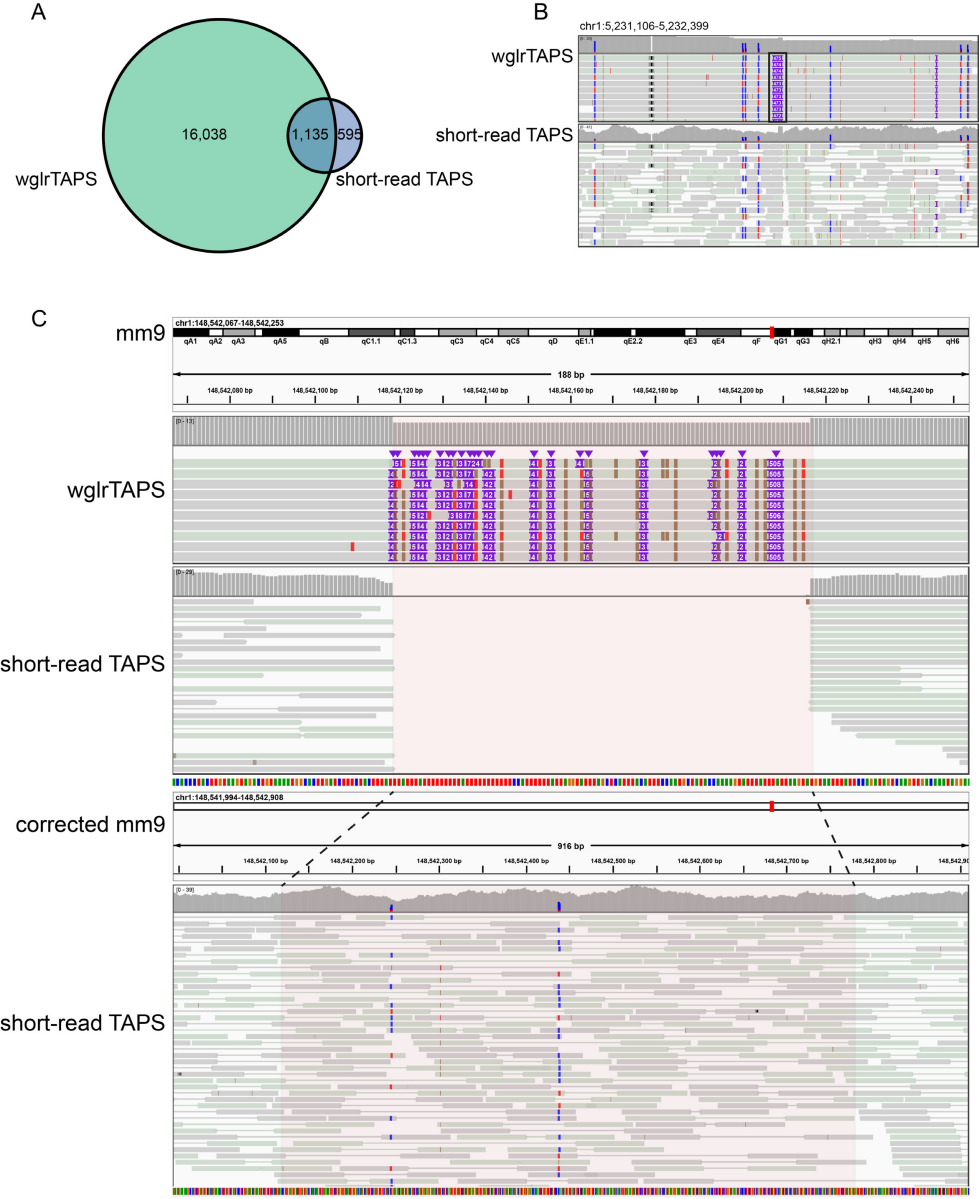
Insertion detection using short-read TAPS and wglrTAPS. (**A**) Venn diagram showing the number of insertions detected in wglrTAPS alone, both wglrTAPS and short-read TAPS, or short-read TAPS alone. (**B**) Example of insertion only detected in wglrTAPS visualized using IGV in bisulfite mode with CG option. The blue colour denotes converted cytosine, and the red colour denotes unconverted cytosine. The insertion is shown in the box. (**C**) The upper panel showing a region only covered in wglrTAPS but not in short-read TAPS. Lower panel showing re-aligned short-read TAPS reads after using the reference corrected by wglrTAPS. The tracks were visualized using IGV in bisulfite mode with CG option. The blue colour denotes converted cytosine, and the red colour denotes unconverted cytosine.

Long-read sequencing is known to improve SV detection (35). We utilized cuteSV (31) and manta (32) to call SV for wglrTAPS and short-read TAPS, respectively, and compared the SVs detected within wglrTAPS and short-read TAPS (Supplementary Figure S3A). To mitigate the effect of algorithm difference, we only included the most operationally defined SV (>50 bp) for comparison (36). Considering the possible effect of sequencing depth on SV detection, short-read TAPS reads were further downsampled to the same depth as wglrTAPS for comparison. As shown in Supplementary Figure S3A, wglrTAPS could detect more SV compared to non-downsampled short-read TAPS and even much more SV compared to downsampled short-read TAPS. These results are consistent with previous findings that PacBio long-read sequencing is more sensitive in resolving SV than short-read sequencing (37), especially for deletions and insertions. Among the SVs, insertions and deletions have been reported as the most commonly studied SVs (38,39). When compared with the non-downsampled short-read TAPS, a total of 17 173 and 1730 insertions were called in wglrTAPS and short-read TAPS, respectively, of which 1135 are overlapping (Figure 3A and B). A low sensitivity in detecting insertions was observed for short-read TAPS, which was consistent with the previously reported historical difficulty of short-read data in genotyping insertions (15,37). In terms of deletion events, we identified 22 900 and 17 374 deletions in total derived from wglrTAPS and short-read TAPS, respectively, with 10 264 overlapping deletions (Supplementary Figure S3B and C). This observation is in agreement with a previous study suggesting that short-read biases towards deletions rather than insertions (40). Upon further analysis on the deletions and insertions only covered in wglrTAPS, we found that a great majority of them overlapped with repetitive regions of the genome (Supplementary Figure S3D and E), which agrees with the previous report that short-read sequencing has difficulty in resolving structural variants, particularly within repetitive regions (37,41). Figure 3C shows a region that cannot be covered by short-read TAPS due to multiple insertion events. Note that not only can wglrTAPS alignments correct the assembly errors, but more importantly two modified CpG sites were detected within the patched loci (Figure 3C). This observation highlights the potential of applying wglrTAPS to investigate the methylation within SVs and repetitive regions.

### Phasing allele-specific methylation using wglrTAPS

**Figure 4.**
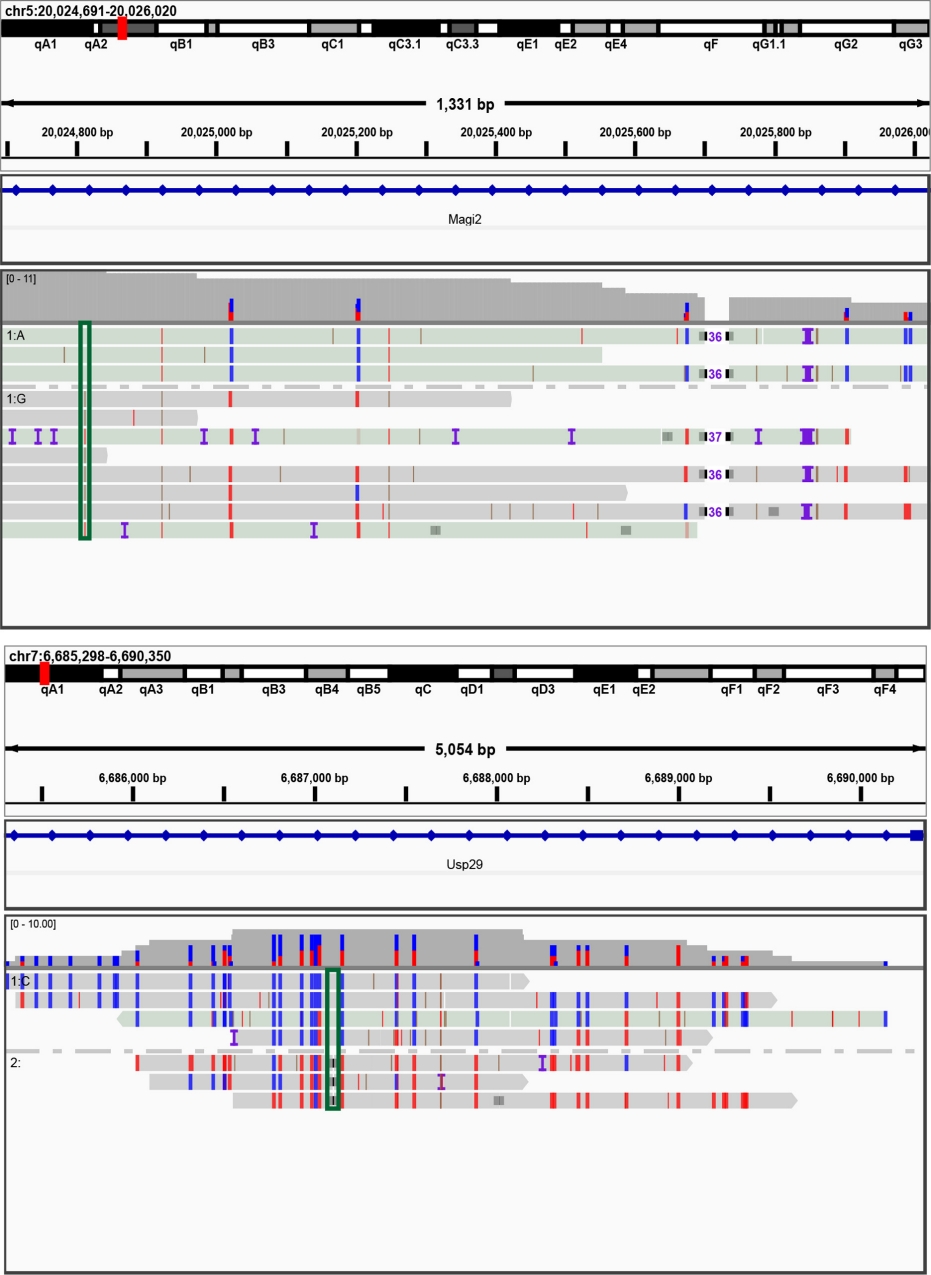
Detecting allele-specific methylation using wglrTAPS. IGV snapshot showing the reads from wglrTAPS aligned to two imprinting genes. The heterozygous SNP (A to G) and deletion are shown in the box. The tracks were visualized using IGV in bisulfite mode with CG option. The blue colour denotes converted cytosine, and the red colour denotes unconverted cytosine.

Simultaneous profiling of allele-specific single-nucleotide polymorphisms (SNPs) and DNA methylation is necessary to detect allele-specific methylation. While whole-genome BS-seq is the most commonly used method, it remains challenging for short-read methods to resolve haplotyped methylomes (42). Long reads across several kilobases can overcome the requirement of a high SNP density, offering a great opportunity for such analysis. To evaluate whether the allelic epigenetic marks can be detected by wglrTAPS, we attempted to combine methylation and haplotyping data to phase possible allele-specific methylation events. It is known that allele-specific DNA methylation is regarded as a hallmark of imprinted genes (43). We herein restricted our attention on previously reported imprinting genes in mice and found clear allele-specific methylation patterns on Magi2 (44) and Usp29 (45) (Figure 4). These were not resolved using short-read TAPS because of the low SNP density and the limited read length. The ability of wglrTAPS to obtain long-range PCR amplicons at a whole-genome scale thus facilitates in-depth study of epigenetic phasing, making it an informative new method for the determination of allele-specific methylation.

## DISCUSSION

As a non-destructive method, TAPS can preserve longer DNA fragments, enabling accurate long-range DNA methylation and hydroxymethylation sequencing. Building on the mild TAPS reaction, we have developed wglrTAPS, a novel approach for whole-genome long-read methylome analysis by combination with PacBio long-read sequencing technology. Unlike the nanoEM method of converting unmodified cytosine to thymine, wglrTAPS offers direct long-range methylation profiling across the whole genome at base resolution.

Using mESC gDNA as an example, wglrTAPS library can easily be prepared, and the resulting long PCR amplicons were sequenced using PacBio Sequel IIe. A total of 6.5 million reads of 3.5 kb N50 length were generated. The obtained sequencing data can be analysed using the standard long-read aligner—minimap2, thus circumventing the need for a new mapping algorithm, as required by nanoEM (26). Moreover, with only 8.2× sequencing depth, the correlation coefficient between short-read TAPS and wglrTAPS was still as good as 0.68 at individual CpG level. We demonstrated that wglrTAPS could enable methylation profiling on complex regions with repetitive elements or SVs, which short-read TAPS could not resolve. In addition, long-read methylome analysis could be used to phase the epigenetic variations on long single DNA molecules. Indeed, by analysing long-read sequencing data, we showed that wglrTAPS is capable of dissecting allele-specific methylation in imprinting genes. wglrTAPS can also inform the methylation status within repetitive regions (Figure 2C), which was missed by short-read TAPS. These significant discoveries demonstrate that wglrTAPS could be widely applicable to other clinical materials, since it is known that aberrant DNA methylation in long repetitive elements and imprinting regions is associated with human diseases such as cancer (2,46,47). Considering the cost of long-read sequencing, we envisage that by combining low-depth long-read sequencing and deep Illumina short-read sequencing it would be possible to deliver more comprehensive epigenetic information at a genome-wide scale. Future work focused on improving the long-range PCR efficiency to generate longer library could further reduce the cost of long-read sequencing. In addition, further development of modified wglrTAPS (wglrTAPSβ and wglrCAPS) (48) holds the potential to distinguish whole-genome 5mC and 5hmC specifically, offering an opportunity to further examine the function of these two epigenetic modifications over long genomic distances.

## DATA AVAILABILITY

All sequencing data are available in SRA under BioProject PRJNA803973. The code used to process whole-genome long-read TAPS data can be downloaded from https://github.com/jfeicheng92/wglrtaps. The code is available under the MIT license.

## Supplementary Material

gkac612_Supplemental_File

## Notes

Present address: Yibin Liu, Exact Sciences Innovation, The Sherard Building, Oxford OX4 4DQ, UK.
