# Supplementary material for: Whole-genome long-read TAPS deciphers DNA methylation patterns at base resolution using PacBio SMRT sequencing technology
Source: Nucleic Acids Res. 2022 Jul 18;50(18):e104. doi: 10.1093/nar/gkac612 (PMC9561279; doi:10.1093/nar/gkac612)
Supplement: gkac612_Supplemental_File [file gkac612_supplemental_file.pdf]

Supplementary Data

Whole-genome long-read TAPS deciphers DNA methylation patterns at base resolution using PacBio SMRT sequencing technology

Jinfeng Chen[1,2,†], Jingfei Cheng[1,2,†], Xiufei Chen[1,2], Masato Inoue[1,2], Yibin Liu[1,2,3], Chun-Xiao Song[1,2]*

[1] Ludwig Institute for Cancer Research, Nuffield Department of Medicine, University of Oxford, Oxford OX3 7FZ, UK
[2] Target Discovery Institute, Nuffield Department of Medicine, University of Oxford, Oxford, OX3 7FZ, UK
[3] Present Address: Yibin Liu, Exact Sciences Innovation, The Sherard Building, Oxford, OX4 4DQ, UK

† These authors contributed equally to this work.

* Corresponding author. Email: chunxiao.song@ludwig.ox.ac.uk (C.-X.S.).

Supplementary figures and table

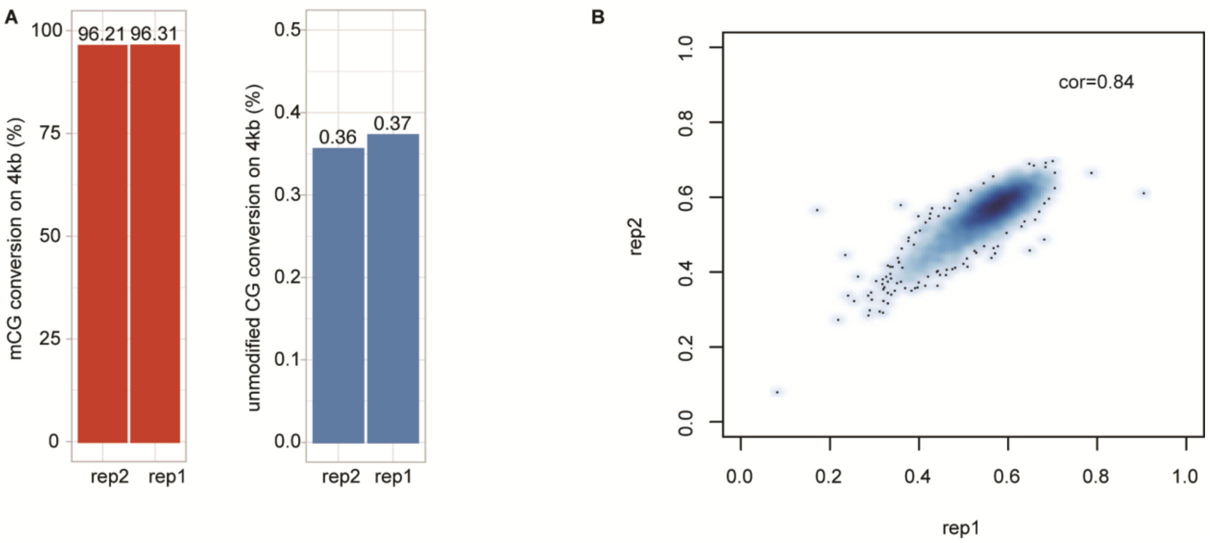

Supplementary Figure S1. Reproducibility validation of whole-genome long-read TAPS (wglrTAPS).

A. Conversion rate of wglrTAPS at methylated CpG sites and false-positive rate of wglrTAPS at non-methylated CpG sites from C^{m}CGG-methylated 4 kb spike-in in two technical replicates.

B. Scatterplot showing the correlation analysis between rep1 and rep2 in 500 kb bins. Pearson's correlation coefficient is shown on the top right.

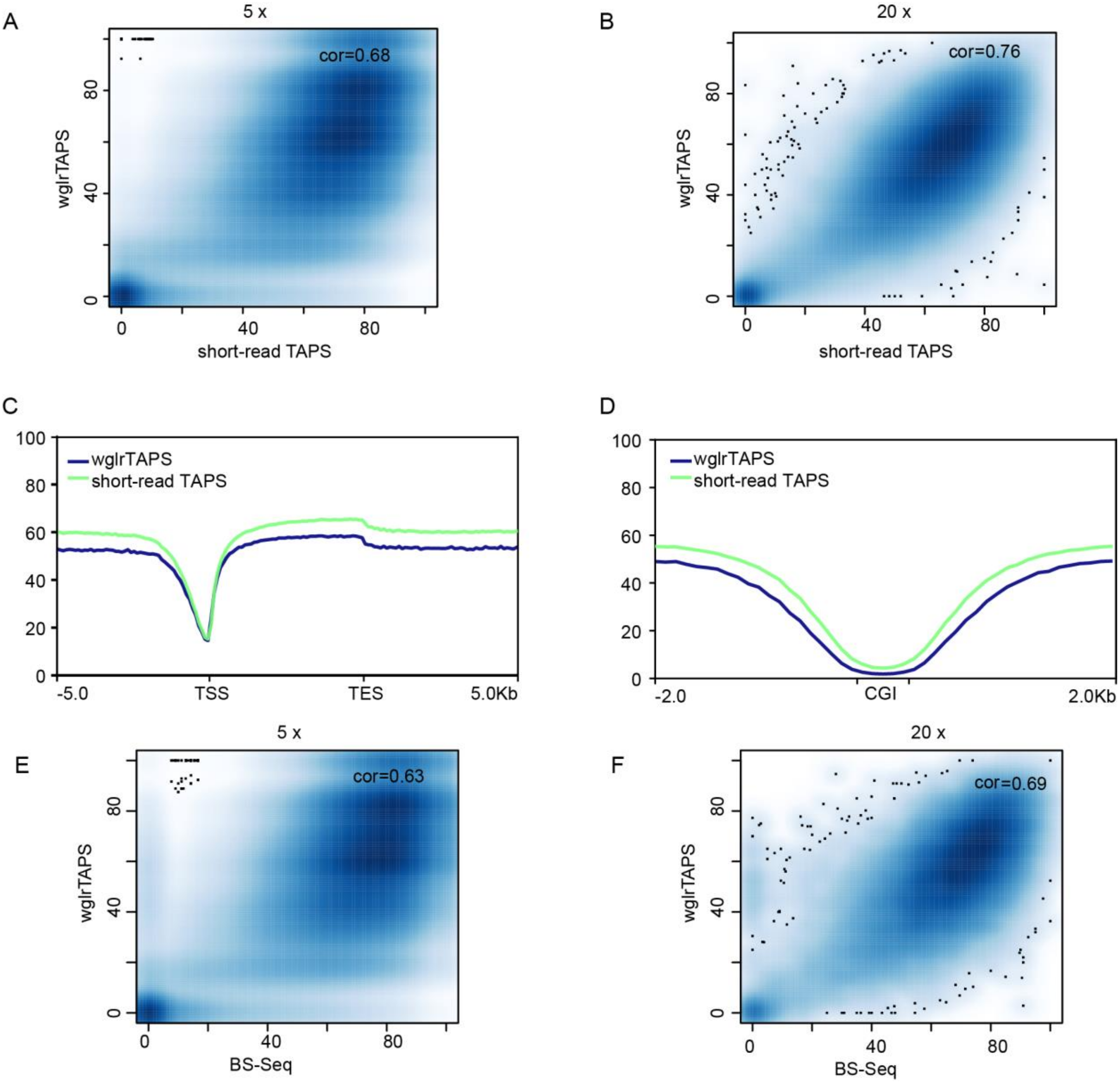

Supplementary Figure S2. Methylation profiling using short-read TAPS and wglrTAPS.

A-B. Scatterplot showing the correlation analysis between wglrTAPS and short-read TAPS at CpGs with the minimal depth of 5x (A) and 20x (B), Pearson's correlation coefficient is shown on the top right.

C-D. Average methylation distribution around gene (C) and CGI (D) in wglrTAPS and short-read TAPS. TSS: transcription start sites; TES: transcription end sites.

E-F. Scatterplot showing the correlation analysis between wglrTAPS and BS-seq at CpGs with the minimal depth of 5x (E) and 20x (F), Pearson's correlation coefficient is shown on the top right.

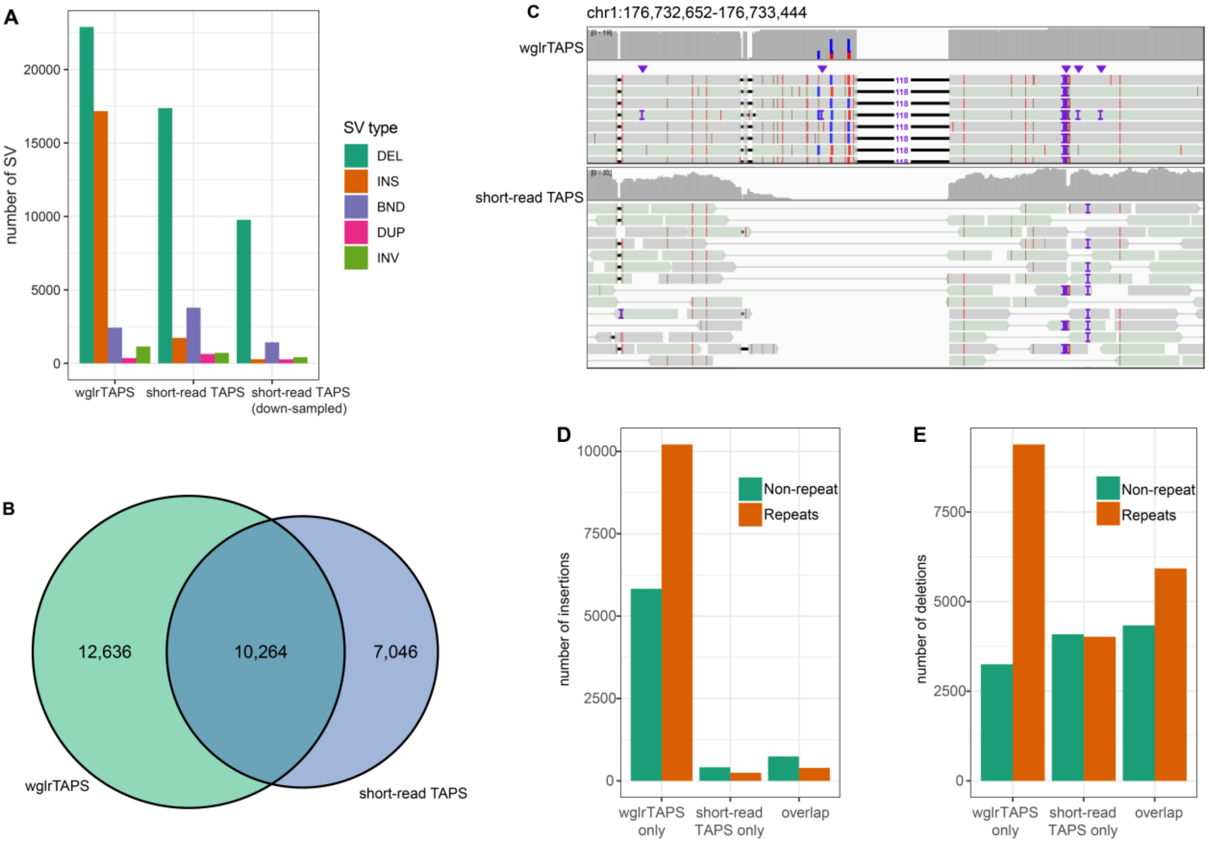

Supplementary Figure S3. Deletion detection using short-read TAPS and wglrTAPS.

A. Bar plot showing the number of SV detected in both wglrTAPS, short-read TAPS and down-sampled short-read TAPS. DEL: Deletion; INS: Insertion; BND: Break end; DUP: Duplication; INV: Inversion.

B. Venn diagram showing the number of deletions detected in wglrTAPS alone, both wglrTAPS and short-read TAPS, or short-read TAPS alone. Deletions detected in wglrTAPS were used as reference. Among the overlapped deletions, there are 61 deletions in wglrTAPS overlapped with multiple deletions in short-read TAPS.

C. IGV snapshot showing an example of deletion that was only detected in wglrTAPS.

D-E. Bar plot showing the number of insertions (D) and deletions (E) that overlap with repetitive regions in the mouse genome.

Supplementary Table S1. Primers and Y-shaped adapter used for wglrTAPS

Template	Primer/Adapter	Sequence (5' to 3')	Note	Ta
4 kb spike-in control	4 kb-F1	ACTGGAACAACACTCAACCCTA	Amplicon size 4280 bp	62ºC
	4 kb-R1	AGGGTGGTGAATGTGAAACC		
4 kb spike-in (validation of TAPS conversion)	4 kb-F2	CATCGAGCATCAAATGAAACTGC	Amplicon size 4012 bp	60ºC
	4 kb-R2	ACGTTATACGATGTCGCAGAGT		
mESC gDNA (for wglrTAPS)	wg-primer1	CACATATCAGAGTGCGCCGAGATCTAC ACTCTTTCCCTACACGAC (Highlight: barcode)	Whole-genome	60ºC
	wg-primer2	CACATATCAGAGTGCGTACGAGATACA TCGGTGACTGGAGTTCAGACGTGT (Highlight: barcode)		
Y-shaped adapter (IDT, HPLC purified)	Two oligos	AATGATACGGCGACCACCGAGATCTAC ACTCTTTCCCTACACGACGCTCTTCCGA TCT	–	–
		GATCGGAAGAGCACACGTCTGAACTCC AGTCAC CGATGTATCTCGTATGCCGTCTTCTGCT TG		
